# P-718. Sexual Networks of Military Service Members with Chlamydia

**DOI:** 10.1093/ofid/ofaf695.930

**Published:** 2026-01-11

**Authors:** Cecelia Peden, Scott Maddox, Nancy Strahan, Tamico Stubblefield, Yezenia Cadena-Malek, Cynthia Bell, Eduardo MendezLanda, Joseph Marcus

**Affiliations:** Uniformed Services University of Health Sciences, Bethesda, MD; Brooke Army Medical Center, DHA, San Antonio, Texas; Army Public Health Nursing, San Antonio, Texas; Army Public Health Nursing, San Antonio, Texas; Department of Public Health, San Antonio, Texas; Army Public Health Nursing, San Antonio, Texas; Army Public Health Nursing, San Antonio, Texas; Brooke Army Medical Center, San Antonio, TX

## Abstract

**Background:**

In order to prevent transmission of sexually transmitted diseases (STIs), public health systems must create policies that are based on both the prevalence of infections as well as sexual networks. In the United States military there are limited data regarding sexual networks, which makes designing strategies to combat STIs challenging. In this project, we describe reported sexual networks of military service members with chlamydia to inform future interventions to decrease transmission.

**Table 1:**
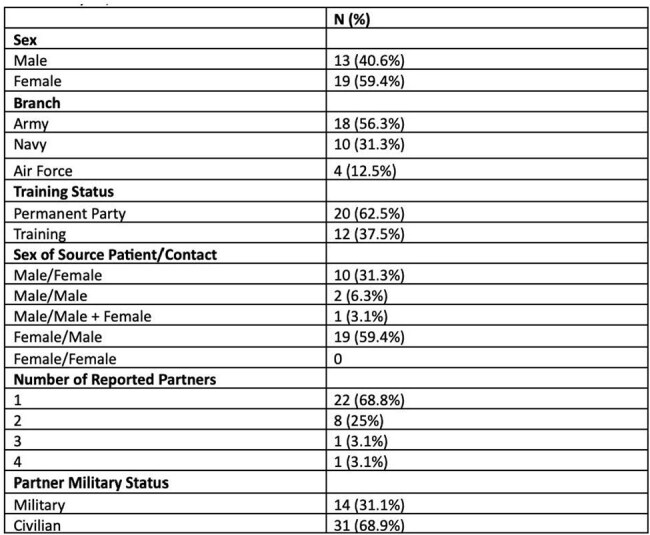
Demographic information of 32 active-duty service members at Joint Base San Antonio with chlamydia, June-December 2023

**Table 2:**
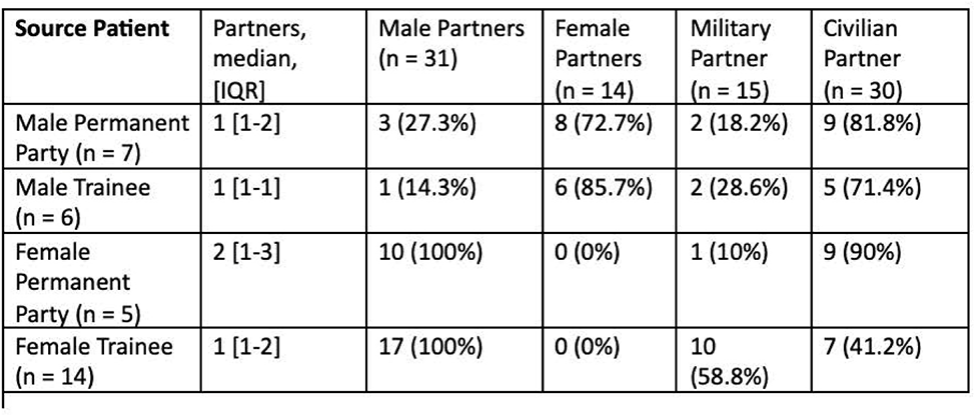
Sexual Networks of Military Service Members with Chlamydia by Sex and Training Status

**Methods:**

Military service members who tested positive for chlamydia at Joint Base San Antonio between June-December 2023 were asked to identify their partners for the preceding sixty days via routine Army Public Health Nursing (APHN) contact tracing. Responses were anonymized to compare sexual networks of patients by sex, branch, and whether they completed all military training (permanent party) or were in a training status.

**Results:**

Thirty-two active-duty service members were identified by APHN with positive chlamydia testing during the study period. Patients with chlamydia were predominantly female (59.4%), in the Army (56.3%), and had completed their military training (62.5%) (Table 1). Of the 45 sexual contacts identified, the majority (66.7%) were civilians. Those still in military training were more likely to have sexual contacts who were also military service members as compared permanent party (50% vs. 14.3%, p=0.014) (Table 2).

**Conclusion:**

This project identified that sexual networks for service members who developed chlamydia were more likely to include military partners when the service member was in a training status than after a service member completed training. This data should inform the development of local prevention efforts that focus on sexual health and disrupt transmission events.

**Disclosures:**

All Authors: No reported disclosures

